# A Robust and Accurate Filter Paper–Based Dried Plasma Spot Method for Bictegravir Monitoring in HIV Therapy

**DOI:** 10.1002/rcm.10110

**Published:** 2025-07-24

**Authors:** Arpita Sathyanarayanan, Roopashri N. Arekal, Divyashree Somashekara

**Affiliations:** ^1^ Department of Biotechnology Manipal Institute of Technology, Manipal Academy of Higher Education Udupi Karnataka India; ^2^ Department of Microbiology, Biotechnology and Food Technology Bangalore University Bengaluru India

**Keywords:** bictegravir, chromatography, dried plasma spot (DPS), therapeutic drug monitoring (TDM)

## Abstract

**Context:**

Therapeutic drug monitoring (TDM) involves the collection of biological samples such as blood, plasma, urine, and saliva. The most commonly used biological matrix for the detection of drugs is either blood or plasma, as they are widely accepted by the regulatory authorities. Such studies require a significant amount of blood to be collected and even more if the study is performed in a plasma sample. The growing demand to minimize the blood or biological samples required for the study of drugs, dried blood spot, or the dried plasma spot techniques has been studied by its demand.

**Objective:**

The main aim was the development of a novel method for the determination of the circulating blood plasma levels in clinical samples using spotted and dried plasma on filter paper as a substrate detection of bictegravir, an HIV integrase strand transfer inhibitor (INSTI) drug from dried plasma spots.

**Materials and Methods:**

The quantitation, as well as the detection of the plasma drug concentration, was done using liquid chromatography–tandem mass spectrometry LC‐MS/MS. Sixty microliters of plasma spiked with 2% of the drug was spotted on Whatman filter paper and was left to dry at room temperature. The drug was extracted using methanol as a precipitating agent.

**Results:**

The extraction technique yielded a recovery of 100%. The assay exhibited excellent linearity in the range of 20–1200 ng/mL.

**Discussion and Conclusion:**

The method developed is a robust, simple, and accurate method to extract drug from the plasma. This method enables to produce a clean sample, proving to be cheaper and more accurate with maximum recovery.

## 1 Introduction

Therapeutic drug monitoring (TDM) is the clinical practice of measurement of drug concentration in different biological matrices in the presence of endogenous compounds naturally existing in the blood [1, 2]. The most important factors in drug discovery and monitoring of the drug candidate are the study of drug disposition in the body. This involves the absorption, distribution, metabolism, and excretion of the drug in the body. This process is a complex process that requires the study of the concentration of the drug in the biological samples after subjecting the candidate to the drug. It is also known that drugs, after administration, are majorly found in the blood or blood plasma, depending on the drugs' affinity and its physicochemical properties [3, 4]. TDM involves the collection of biological samples such as blood, plasma, urine, and saliva. The most used biological matrix for the detection of drugs is either blood or plasma, as they are widely accepted by the regulatory authorities. Such studies require a significant amount of blood to be collected and even more if the study is performed in plasma samples. The growing demand to minimize the blood or biological samples required for the study of drugs, dried blood spot, or the dried plasma spot (DPS) techniques has been studied by its demand.

Despite the growing use of DPS techniques in TDM and bioanalysis, several research gaps remain unaddressed. One significant limitation is the lack of standardized protocols for sample collection, drying, and storage, which can lead to variability in analyte recovery and stability. Furthermore, the influence of hematocrit‐independent plasma separation methods on quantification accuracy is not fully understood, particularly for drugs with high protein binding like bictegravir. The limited data on the long‐term stability of analytes under varying environmental conditions (humidity and temperature) and the potential for matrix effects such as ion suppression during mass spectrometric analysis further complicate the method's reliability. Additionally, most existing studies focus on method validation under controlled conditions, with fewer investigations into real‐world clinical applicability, cross‐contamination risks, or scalability for high‐throughput screening. Addressing these gaps is essential for broader adoption and regulatory acceptance of DPS in clinical and pharmacokinetic studies.

Because of the innovation of the micro sampling technique, the amount of plasma required in the preparation of a dried plasma spot is as little as 50–60 μL, as opposed to 300–400 μL required in conventional extraction techniques [3, 5]. The procedure for the extraction of drugs from dried plasma or blood spots has been applied in the study of other drugs, such as Benznidazole [6], Amlodipine, Atenolol, Atorvastatin, Digoxin, Enalapril, Losartan, Propranolol, Simvastatin, and Sulfameter, Fenofibrate, Furosemide, Nifedipine, and Valsartan [7], Paroxetine [8], Dexmedetomidine [9], Teriflunomide [10], Daptomycin [11], [12], and many more including antiviral drugs.

**FIGURE 1:**
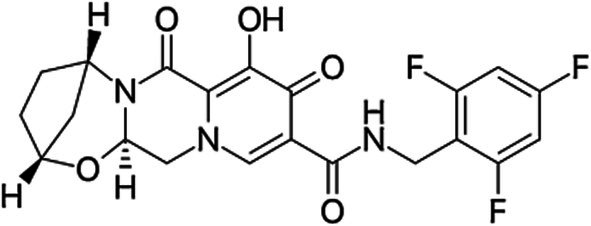
Chemical structure of Bictegravir with a tricyclic pyrrolo [1,2‐a]pyrazine derivative with a hydroxyacetyl side chain, a 2,4‐difluorobenzyl substituent, and a carboxamide group, designed for integrase inhibition via metal ion chelation [13].

Bictegravir is a potent HIV integrase strand transfer inhibitor (INSTI), which is generally administered as a combination drug along with tenofovir alafenamide and emtricitabine. This drug is commercially known as Biktarvy by Gilead Sciences Inc. (Foster City, CA, USA). Bictegravir, along with tenofovir alafenamide and emtricitabine, is indicated as a complete drug regimen used in treating human immunodeficiency virus type 1 (HIV‐1) infection in adults who have an antiretroviral treatment history or who are virologically suppressed. This drug is observed to have high protein affinity and is seen to be bound to plasma protein more than 99%, therefore decreasing its interactions with other drugs, its clearance, and increasing its solubility. Bictegravir is extensively bound to albumin and 1‐acid glycoprotein, making PK/PD analysis imperative (Figure 1) [13].

The calibration range was set based on the available literature. Raju et al. in his findings have tested bictegravir in the range of 5–1500 ng/mL [14]. In another finding done by Gouget et al., the range for calibration was set to 10–5000 ng/mL [15]. Based on the available literature, we decided to set the calibration range from 20 to 1200 ng/mL.

Various methods of detection of bictegravir, a potent HIV integrase strand transfer inhibitor (INSTI) from plasma that has previously been studied and discussed [16, 17], are a few to name. This paper aims at the development of a novel method of sample collection—bictegravir—from dried plasma spots. This study also aims at the development of a quantitation method for the drug—bictegravir. This method of microsampling is perhaps the first study reported that uses 60 μL of plasma for the study of this drug, while maintaining the sensitivity as well as selectivity required for the analysis of this drug.

## 2 Materials and Methods

### 2.1 Chemical and Reagents

Bictegravir, bictegravir‐D5 (internal standard), methanol (HPLC grade), formic acid (LR grade or higher grade), ammonium formate (LR grade or higher grade), water (Barnstead/Milli‐Q water), human plasma–K_2_EDTA, centrifuge, sonicator, API‐3200 Q trap mass spectrometer with Shimadzu HPLC, multiple vortexer, polypropylene vials and polypropylene tubes, Whatman filter paper 42, Whatman filter paper 1, micropipettes and multipipettes, deep freezer and refrigerator, and measuring apparatus.

### 2.2 Preparation of Standards and Stock Solutions

The concentration of the bictegravir stock solution was 1.5 mg/mL. This was prepared by weighing 15.0 mg of drug working standard in 10 mL of methanol. The resultant mixture was vortexed to ensure proper mixing. This solution was stored at 2°C–8°C until further use. The concentration of the internal standard drug stock solution was 0.10 mg/mL. This was prepared by weighing 0.526 mg of the working standard of internal standard in 5 mL of methanol. The resultant mixture was vortexed to ensure proper mixing. This solution was stored at 2°C–8°C until further use.

The CC values that are the standard curve values were prepared with the nominal concentration as 12 054.688, 10 246.485, 8422.610, 6022.166, 1812.672, 605.432, 40.362, and 20.181. The QC values that are the HQC, MQC, LQC, and LOQ QC ranged from 9750.890, 4826.691, 60.334, and 20.272. The preparations of the calibration curve and quality control samples for the drug were prepared as explained in Table 1.

**TABLE 1:** Preparation of calibration curve and quality control curve (ng/mL).

Standard	Stock conc. (ng/mL)	Stock vol. (mL)	Total vol. (mL)	Final conc. (ng/mL)	Final plasma concentration after 2% spiking (ng/mL)
STD 8	1 499 339.270	4.02	10.00	602 734.387	12 054.688
STD 7	602 734.387	8.50	10.00	512 324.229	10 246.485
STD 6	512 324.229	8.22	10.00	421 130.516	8422.610
STD 5	421 130.516	7.15	10.00	301 108.319	6022.166
STD 4	301 108.319	3.01	10.00	90 633.604	1812.672
STD 3	90 633.604	3.34	10.00	30 271.624	605.432
STD 2	30 271.624	1.00	15.00	2018.108	40.362
STD 1	2018.108	5.00	10.00	1009.054	20.181
HQC	1 500 136.950	3.25	10.00	487 544.509	9750.890
MQC	487 544.509	4.95	10.00	241 334.532	4826.691
LOC	24 133.453	1.25	10.00	3016.682	60.334
LOQ QC	3016.682	3.36	10.00	1013.605	20.272

#### 2.2.1 Sample Preparation

For the study, the blank human plasma treated with K_2_EDTA was obtained from healthy volunteers who were not being treated with the drug under study. Plasma was obtained from peripheral blood collected in K_2_EDTA‐containing tubes by centrifuging at 4000 rpm (RCF‐3512g) for 5 min and was stored at −30°C until the study. For the study, the calibration curve and quality control were prepared by spiking 2% of the drug of the respective concentration into human plasma. (The plasma samples were deidentified before being supplied to the research laboratory for analysis.)

#### 2.2.2 Dried Plasma Spot Preparation

**FIGURE 2:**
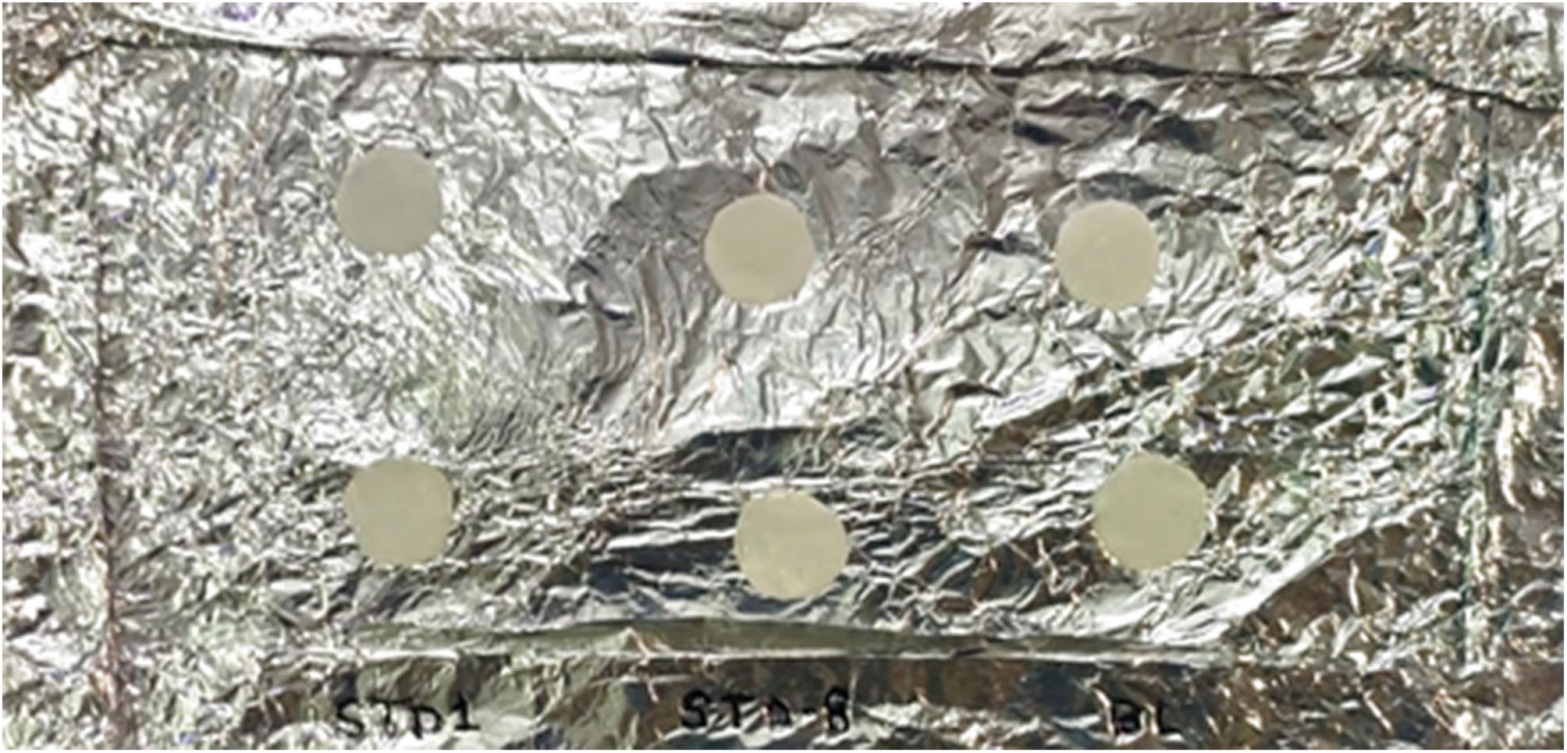
Filter paper with spiked plasma left to dry at room temperature.

To prepare dried plasma spots, Whatman filter paper was cut into circular discs of 1 cm in diameter. This was placed on an aluminum foil sheet, as shown in Figure 2. This was followed by the spotting of 60 μL of spiked plasma on the punched discs, as shown in the image below. These discs were dried at room temperature and stored at 2°C–8°C until further study.

### 2.3 Methods of Extraction

#### 2.3.1 Method of Extraction From Plasma

A plasma aliquot (60 μL) of spiked plasma samples was mixed with 50 μL of ISTD working solution (0.1 mg/mL) into a polypropylene vial. Extraction of the drug and its ISTD from plasma was performed by the protein precipitation technique. Protein precipitation from the plasma was brought about by the addition of 500 μL of methanol to the spiked sample. The resultant solution was then vortexed to ensure mixing. This was followed by centrifuging the sample for 15 min at 5°C at 4645 *rcf*. The supernatant was separated by transferring it to HPLC glass vials and was injected into the LC‐MS/MS system.

#### 2.3.2 Method of Extraction From Dried Plasma

The extraction of the drug from the filter paper was performed by the protein extraction technique. The dried plasma spot prepared as mentioned before was placed in labeled polypropylene vials, and 50 μL of ISTD working solution (0.1 mg/mL) was added to each vial. This was followed by the addition of 500 μL of methanol and was incubated for 15 min to rehydrate the surface of the paper as well as increase the interaction time of methanol with the drug. This was vortexed for 5 min to ensure proper mixing of methanol. The samples were then centrifuged for 15 min at 5°C at 4645 *rcf*. The supernatant thus obtained was transferred to an HPLC glass vial and was injected into the LC‐MS/MS system.

### 2.4 Chromatographic Conditions

The HPLC system used was Shimadzu SIL‐HTC. The chromatography was performed on a Hypersil Gold column with dimensions of 100 × 4.6 mm, 5 μ particle size. The column oven temperature was maintained at 40.0 ± 1.0°C, and the autosampler temperature was maintained at 4.0 ± 1.0°C. The mobile phase was composed of 650 mL of methanol and 350 mL of 0.1% formic acid in water. The final ratio was 65:35, v/v. The selected mobile phase composition of 65:35 methanol:0.1% formic acid in water was optimized for both sensitivity and peak shape for bictegravir (BIC). Methanol was preferred over acetonitrile due to superior ionization efficiency and lower background noise under positive ESI conditions for BIC. Preliminary trials using acetonitrile‐based systems (e.g., ACN:water 60:40 with 0.1% FA) resulted in broader peaks and reduced signal intensity. These findings are consistent with published methods for integrase inhibitors.

The flow rate was set at 1.000 mL/min with an injection volume of 2.00 μL. The total run time was maintained at 3 min. The rinsing volume was maintained at 500 μL with a dip time of 5 s both before and after aspiration.

### 2.5 Mass Spectrometric Conditions

The instrument used for detection is an AB Sciex API 3200 Q Trap Mass spectrometer. The samples were run in positive multiple reaction mode with an electrospray ionization (ESI) source. The source parameters were optimized to get maximum response. The tuning for the same was performed using a 200‐ng/mL aqueous vial. The best analyte response was observed when the CAD, medium; source temperature, 550°C; ion spray voltage, 5000 V; nebulizer (GS1), 45 psi; and drying (GS2), 65 psi gas pressure parameters. The sample loading and the values were controlled manually by Analyst 1.6.3 software.

### 2.6 Statistical Evaluation

The chromatograms have to be acquired using the computer‐based Analyst software 1.6.2. The concentration of spiked human plasma was calculated by following the regression equations with the reciprocal of the drug concentration. The weighting factor is 1/*x* ^2^ for *y* = *mx* + *c*, where *y* is the ratio of the analyte peak area and internal standard peak area (drug area/ISTD area), *x* is the ratio of analyte concentration and internal standard concentration, *m* is the slope of the calibration curve, and *c* is the *y*‐axis intercept value.

## 3 Results

### 3.1 Mass and Chromatographic Parameters

Upon optimization of chromatographic parameters, it was observed that maximum chromatographic separation for the drug was observed at a retention time (RT) of 2.27 min, as well as the postcolumn infusion studies, suggesting that the given chromatographic conditions showed a well‐separated suppression zone and the analyte peak. The RT of 2.27 min was confirmed to be sufficient for analyte separation from matrix interferences. Bictegravir's high protein binding (> 99%) does not impact chromatographic separation but may affect extraction recovery, which was addressed in method validation. Selectivity was assessed by analyzing six blank plasma samples, which showed no endogenous interference at BIC's RT.

**FIGURE 3:**
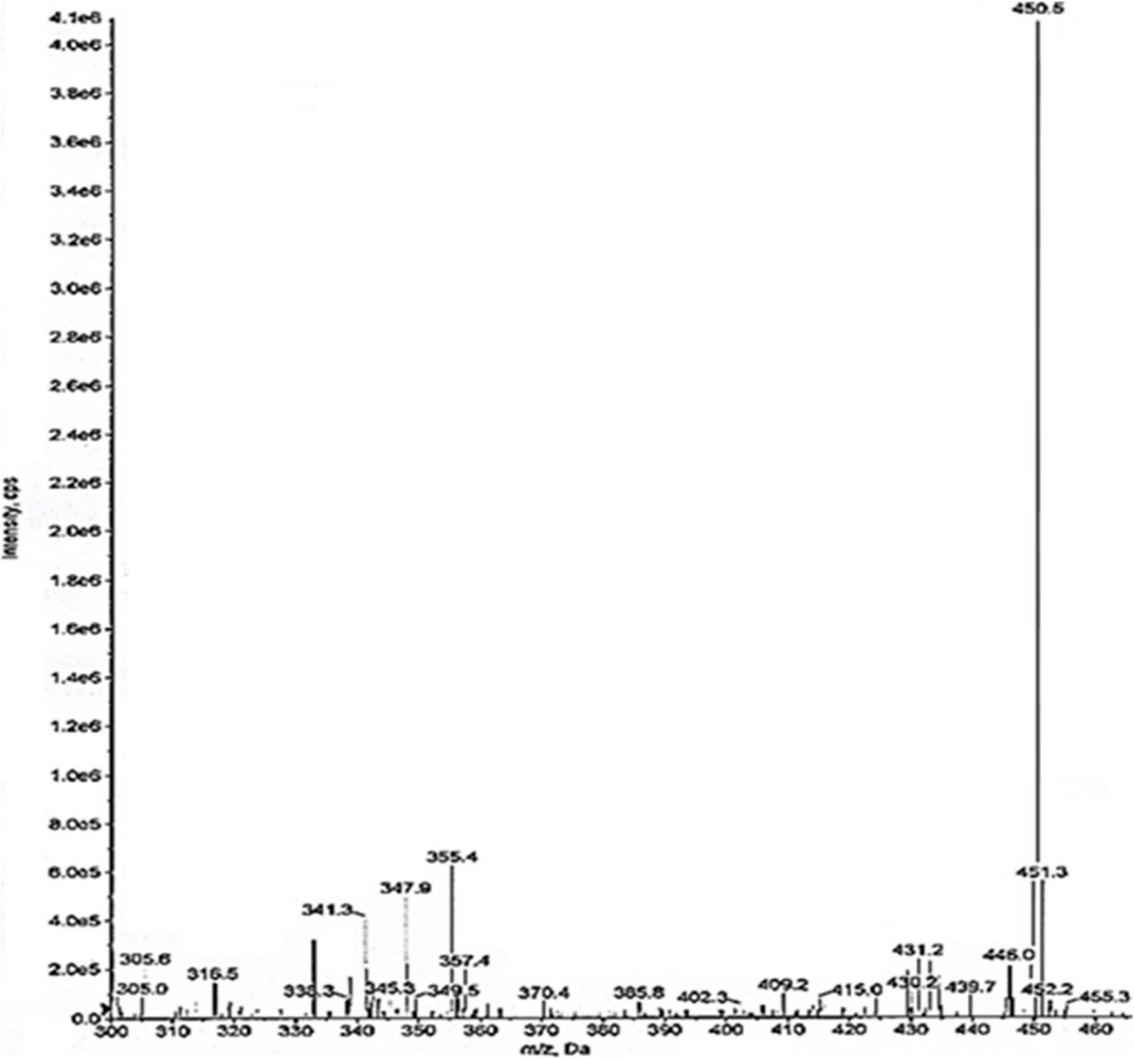
Q1 scan of bictegravir.

**FIGURE 4:**
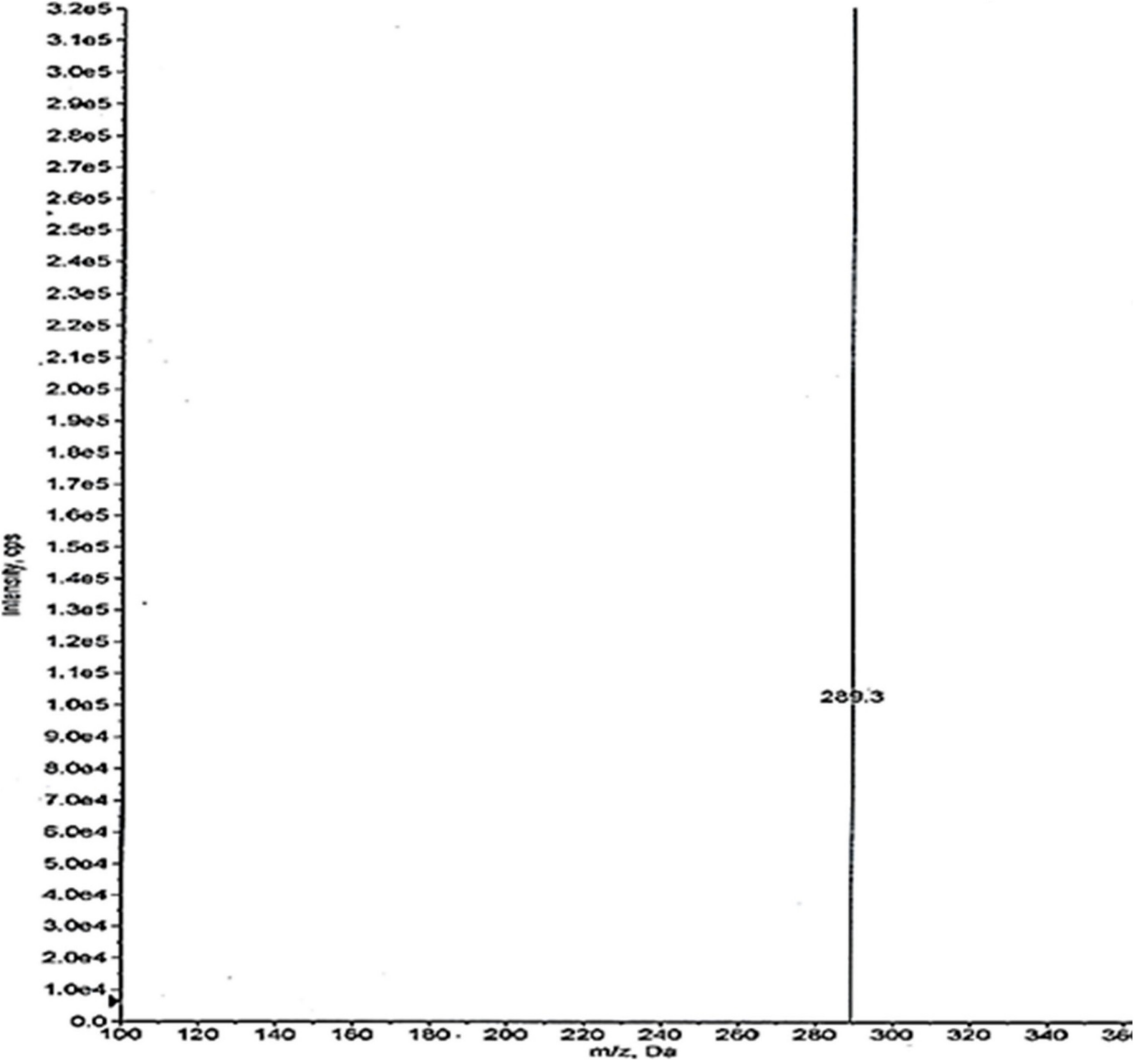
Q3 scan of bictegravir.

Development of mass parameters required for a multiple reaction mode. This was set by loading a 200‐ng/mL aqueous vial. The Q1 and Q3 for the drug were found to be 450 and 289.100. The scans of the same are represented in Figures 3 and 4, respectively.

### 3.2 Optimization of Filter Paper Conditions

A short recovery study was performed to check the recovery of the extraction technique. Trials were taken on two filter papers, which were Whatman filter paper 1 and Whatman filter paper 42 in triplicates (*n* = 3) for the standard 8 samples, as explained in Table 2.

**TABLE 2:** Trial design and result of optimization of filter paper conditions and recovery study.

Filter paper details	Analyte STD‐8 mean area response	Mean area response	ISTD area response	ISTD area response	Mean recovery
Whatman filter paper 42	840 196	803 496.333	163 437	166 273.333	87.22%
	785 042		170 296		
	785 251		165 087		
Whatman filter paper 1	924 705	954 740.333	164 761	161 176.667	103.64%
	966 966		159 993		
	972 550		158 776		
Precipitation	708 231	775 803.333	164 722	158 637.667	84.21%
	790 249		152 983		
	828 930		158 208		
Aqueous vial of STD‐8	921 204	—	149 868	—	—

### 3.3 Recovery

The recovery obtained through the filter paper method was compared against the precipitation technique as well as with the aqueous vial. This was prepared by aliquoting 60‐μL drug standard 8 with 50‐μL internal standard and 500‐μL precipitating solvent. The samples were injected and run under the chromatographic and mass parameters as mentioned above. The mean values of peak area response obtained are listed in the table below. The area response for the aqueous vial was found to be 921 204 for the analyte and 149 868 for the ISTD.

From this study, it was observed that the recovery of the drug through the filter paper method showed nearly 90%–100% recovery, and the percent recovery seen in Whatman filter paper was more as compared with the precipitation method. The Whatman filter paper 42 showed a mean peak area response of 803 496.333, and that of Whatman filter paper 1 was observed to be 954 740.333. Upon comparison of the area ratio response of both filter papers, it was observed that the higher mean area response was observed in the standard 8 analyte processed in Whatman filter paper 1. Whatman filter paper 1 was decided to be used for further study, as it showed nearly 100% recovery.

### 3.4 Validation Parameters

#### 3.4.1 Linearity and Calibration Curves

The spiking solutions over the linearity range were prepared by diluting the higher concentration solution serially. The calibration standards were prepared by spiking known concentrations of the drug working solutions in human plasma. Three linearity curves containing eight nonzero concentrations were analyzed, and the values were plotted in the graph against the nominal concentration using Excel. The regression value and the slope were also obtained through this graph and are represented in Table 3.

**TABLE 3:** Precision and accuracy result.

	Calculated concentration P&A‐1	Nominal concentration
BLANK	N/A	0
BLANK + ISTD	N/A	0
STD 01	19.9425 ± 0.16	20.181
STD 02	40.8795 ± 0.98	40.362
STD 03	582.038 ± 36.84	605.432
STD 04	1869.739 ± 45.10	1812.672
STD 05	6089.938 ± 45.94	6022.166
STD 06	8211.448 ± 191.06	8422.610
STD 07	10 286.1 ± 591.98	10 246.485
STD 08	11 942.18 ± 46.20	12 054.688

#### 3.4.2 Precision and Accuracy

Two different precision and accuracy were performed. Each P&A run had one set of extracted calibration curves along with six replicates each of quality control (LLQC, LQC, MQC, and HQC). The concentration of each sample was back‐calculated from CC, interrun precision was calculated as the coefficient of variation (CV), and accuracy was calculated using the percentage ratio of observed to nominal concentration. The values obtained are listed in Table 4.

**TABLE 4:** Extracted recovery, total recovery, and absolute recovery of the drug were calculated from the data obtained by the software and listed in the table above. A, extracted sample; B, unextracted sample (abs. recovery); C, aqueous sample (total recovery).

	LQC response			MQC response			HQC response		
	A	B	C	A	B	C	A	B	C
	3019	3053	2247	195 426	197 111	184 797	351 770	357 130	339 055
	2939	2997	2448	196 947	198 741	177 527	347 590	359 874	325 370
	2895	2923	2358	195 359	200 122	174 375	324 190	354 370	329 392
	3077	3232	2383	193 979	198 348	176 196	323 462	342 580	336 935
	2837	3054	2243	191 152	202 929	177 101	337 012	358 708	328 599
	2723	2802	2299	190 388	195 921	172 369	344 539	353 005	323 276
Mean	2915	3010	2330	193 875	198 862	177 061	338 094	354 278	330 438
SD	127.27	144.27	81.16	2593.44	2454.48	4243.63	12 060.95	6285.64	6291.56
% CV	4.4	4.8	3.5	1.3	1.2	2.4	3.6	1.8	1.9
% Recovery		96.8	125.1		97.5	109.5		95.4	102.3
Mean % CV (abs. rec)	1.1								
Mean % CV (total rec)	10.4								

#### 3.4.3 Total and Absolute Recovery

The total recovery obtained for all the QC levels was in the range of 95%–100%. Similarly, for the absolute recovery, the values were found to be in the range 100%–125%. The percent coefficient of variation for both total and absolute recovery was also found to be within 5%. The values are tabulated in Table 5.

**TABLE 5:** Extracted recovery, total recovery, and absolute recovery of the internal standard were calculated from the data obtained by the software and listed in the table above.

SR NO	Extracted sample	Unextracted sample (abs. recovery)	Aqueous sample (total recovery)
1	330 527	287 076	300 597
2	372 301	319 901	339 763
3	443 200	396 726	345 931
4	326 739	285 201	291 230
5	371 862	323 547	326 262
6	430 098	394 467	352 440
7	305 891	283 147	293 563
8	369 271	320 876	323 085
9	426 206	396 653	352 456
10	305 882	272 757	304 079
11	363 626	319 247	325 328
12	455 348	405 888	358 809
13	321 737	284 219	293 517
14	360 245	329 565	329 365
15	447 730	387 269	334 642
16	332 141	282 508	291 522
17	355 155	318 214	316 838
18	400 286	391 052	323 534
Mean	373 236	333 240	322 387
SD	49 683.07	48 375.08	22 547.94
% CV	13.3	14.5	7.0
% Recovery		112.0	115.8

## 4 Discussion

The technique described here showed adequate precision and accuracy and produced a significant amount of information about the dried plasma spot. In terms of improvement, a dry spot and short run time would be beneficial for the efficient use of this method for preclinical and clinical pharmacokinetic evaluation of bictegravir.

This technique, as compared with the conventional protein precipitation technique, allows little to no endogenous substances postextraction, making the sample cleaner and less hazy and mass spectrometric friendly. Short‐term (24 h, 48 h) and long‐term (1 week, 1 month) stability studies under room temperature and refrigerated (4°C) conditions have now been performed and reported. The recovery remained within 85%–115%, confirming method robustness under typical clinical handling conditions. One major drawback in the dried blood spot drug concentration detection is the involvement of an additional step of centrifugation before preparation of plasma from the blood withdrawn. This problem can also be mitigated by the introduction of newer technologies, such as blood fractional cartridge [2]; also, ready‐to‐use membranes are available to separate plasma.

The HCT effect is greatly ruled by the nature of the drug. Drugs that are highly attracted to the erythrocytes show more affinity towards RBCs. Therefore, under such circumstances, the HCT value becomes an influencing parameter. In order to reduce the influence of the varying HCT concentration, a volumetric blood spot is required, which is difficult to obtain without skillful and trained personnel, especially at home without guidance.

There are many methods developed to determine the plasma concentration of the drug bictegravir, followed by its detection by LC‐MS/MS. In this paper, the method developed is a robust, simple, and accurate method to extract the drug from the plasma. To the best of our knowledge, there have been no studies published stating the use of filter paper discs to store and determine the concentration of bictegravir. This report is the first to state the use of 60 μL of plasma dried on filter paper utilized for the determination of this drug.

The advent in terms of spot drug detection for TDM using LC‐MS/MS has been gaining popularity owing to its extremely sensitive and selective method of detection. The requirement of small sample volumes and precise detection of the molecules has made LC‐MS/MS detection a preferred choice when it comes to TDM–Therapeutic Drug Detection.

## 5 Conclusion

Using filter paper considerably reduced the presence of endogenous substances from the sample; therefore, it produced a clean sample for mass studies. This gives added benefit to process drugs, which calls for precipitation, a method of extraction. Because of the benefit in transportation, storage, and the reduced requirement of blood, this method shows an advantage over the conventional plasma storage techniques. This method could be successfully applied in further studies for routine analysis in pharmaceutical as well as other industrial settings for sample collection and processing in pharmacokinetic studies.

## Author Contributions

**Arpita Sathyanarayanan:** methodology, investigation, writing – original draft. **Roopashri N. Arekal:** data curation, writing – review and editing, formal analysis. **Divyashree Somashekara:** conceptualization, validation, writing – review and editing, supervision.

## Conflicts of Interest

The authors declare no conflicts of interest.

## Peer Review

The peer review history for this article is available at https://www.webofscience.com/api/gateway/wos/peer‐review/10.1002/rcm.10110.

## Data Availability

The data that support the findings of this study are available from the corresponding author upon reasonable request.
